# Evaluation of the Relationship between the Incubation Time and Carotenoid Production in *Rhodotorula Slooffiae* and *R. Mucilaginosa* Isolated from Leather Tanning Wastewater 

**Published:** 2013-10

**Authors:** Farzaneh Sadat Naghavi, Parichehr Hanachi, Mohammad Reza Soudi, Azra Saboora, Atefeh Ghorbani

**Affiliations:** 1 Department of Biology, Faculty of Science, Alzahra University, Tehran, Iran

**Keywords:** Antioxidant, Carotenoid, Incubation time, *Rhodotorula* Spectrophotometry

## Abstract

***Objective(s):*** Carotenoids which are naturally synthesized by fungi such as yeasts can act as an antioxidant which is closely related to their ability to decrease the risk of a variety of degenerative diseases. In recent years, the increase of demand for carotenoids obtained from natural sources has promoted major efforts to improve carotenoid production from biological sources such as pigmented yeasts. The aim of this study was comparing incubation time and carotenoid production in *Rhodotorula slooffiae* and *R. mucilaginosa* isolated from leather tanning wastewater.

***Materials and Methods:*** To isolate the carotenoid pigment, cells were suspended in acetone and broken using a homogenizer, followed by centrifugation and separation of supernatant. In order to study the effect of incubation time, samples were held at 30 ˚С in a shaker at 150 rpm for 24, 48, 72, 96, and 120 hr. For analytical evaluation, pigments were measured spectrophotometrically at 450 nm using the extinction coefficient E^1%^_450_=2500.

***Results:*** The results showed that the content of total carotenoid in *R. slooffiae* was the highest when samples were incubated for 72 hr. Overall, *R. mucilaginosa *had more potential to produce carotenoid*. *The best incubation periods for *R. slooffiae* and *R. mucilaginosa* were 72 hr and 48 hr, respectively.

***Conclusion:*** It seemed that the maximum rate of total carotenoid was not directly associated with the maximum amount of cell biomass and the type of carotenoid and their relative amount may vary depending on genus of yeast.

## Introduction

All body cells are constantly exposed to internal and external oxidants. In recent years, the intensification of air pollution, UV radiation, smoking and improper diet has increased the amount of these oxidants alarmingly and ultimately leads to incidence of various disease and disorders such as cancer, cardiovascular disease and cataracts in human. Carotenoids are the most common pigments in nature which are responsible for the presence of yellow, orange or red color of many foods e.g. fruits, vegetables, etc. Aside from being natural pigments, carotenoids also have important biological activities. 

 It is well known that some carotenoids are precursors of vitamin A (1). The chemical structure of carotenoids (the presence of double bonds) accounts for the abilities of these compounds to perform photosynthesis, photo protection, quenching singlet oxygen and possessing antioxidant properties. The principle function of antioxidants is delaying the oxidation of other molecules by inhibiting the initiation or propagation of oxidizing chain reaction by free radicals and they may reduce oxidative damage to human body (2). This latter property is closely related to their ability to decreased risks for a variety of degenerative diseases such as cancer, cardiovascular disease, macular degeneration and cataract (3-5). 

 Carotenoids are synthesized by all photosynthetic organisms and fungi such as yeast and can be used in animal or human food supplements. Access to natural and cheap resources in order to produce supplements and antioxidant drugs can be a big help in the prevention and treatment of the consequences of the presence of oxidants in body. Traditionally, carotenoids were extracted from plants such as annatto, paprika and saffron, whereas today microbial carotenoids have attracted much attention because of the ease of increasing production by environmental and genetic manipulation. The commercial use of microorganisms with biotechnological potential to produce carotenoids has been successful and pigmented yeast is an interesting subject from this point of view. Among pigmented yeasts, only some small taxonomic groups have been investigated regarding their carotenoid pigments content. Along with the most known producer of carotenoid, *Phaffia rhodozyma*, there is some evidence of the capacity for carotene production by other well-known pigmented yeasts of genus *Rhodotorula* (5, 6). The composition and amount of carotenoid pigments in numerous natural isolates of the genera *Rhodotorula/Rhodosporium* and *Sporobolomyces*/ *Sporidiobolus* were studied in sufficient detail in 1970s (7). The type of carotenoid and their relative amount may vary depending on genus of yeast and environmental condition. Since the pigment production via fermentation depends on culture condition and the genus of microorganism, the process can be managed to produce more carotenoid compounds. Several studies have been done for optimization of different conditions of carotenoids production by the strains of *Rhodotorula *aimed at both increasing and maximizing the production of these pigments (8-11). Optimization of the medium and environmental conditions are necessary for microbial fermentations to fully exploit the potential of the selected microbial strains (12). 

 The aims of the present study were to compare between amount of total carotenoid and evaluating the effect of incubation time on pigmented yeasts previously isolated from leather tanning wastewater.  

## Materials and Methods

***Cultures and cultivation***

The red yeasts strains, isolated from leather wastewater, were obtained from the private culture collection at the National Laboratory of Industrial Microbiology, Alzahra University, Tehran, IR Iran. Out of 7 samples collected, only 2 had a yellow to red color at high intensity which was *R. slooffiae* and *R. mucilaginosa. *Samples were maintained in Petri dishes containing yeast extract-peptone-glucose agar medium** (**YPG agar**) **[g/l]: yeast extract 5, agar 17, peptone 10, dextrose 20, and pH = 6.

***Carotenoids analysis***

Samples were prepared by cultivation of the yeast strain on YPG agar at 30^o^C for 48 hr. transferring one full loop into YPG broth and incubation on a rotary shaker at 150 rpm and 30 ˚C, overnight (13). Main cultures cultivation (Semi-synthetic medium (MMS): [g/l]: glucose 10, [NH_4_]_2_SO_4_ 2, KH_2_ PO_4_ 2, MgSO_4_.7H_2_O 0.5, CaCl_2_.2H_2_O 0.1, yeast extract 1, pH= 5) was carried out in a 500 ml Erlenmeyer flask containing 100 ml of medium inoculated with 3 ml of the suspension which has an initial cell concentration as an optical density of 0.5 at 600 nm. Incubation was performed on a rotary shaker at 150 rpm and 30 ˚C for 72 hr. All experiments were performed in triplicate. After cultivation, cells were harvested by centrifugation at 1×10^4^ rpm for 20 min and washed three-time with distilled water and centrifuged again. The obtained biomass was at first held at -70 ˚С for 24 hr and then was kept at 35 ˚С for 24 hr. The methods of Davis (14) with modification were used for the extraction of carotenoid pigments. Briefly, cells were harvested by centrifugation at 1×10^4^ rpm for 10 min and were washed three times with distilled water. Cells were ruptured three times with 12 ml of acetone and broken using homogenizer (Witeg, Germany). The suspension was then centrifuged and the supernatant was collected. Acetone extracts were pooled in a funnel and carotenoid pigments were extracted twice with an equal volume of petroleum ether. The total carotenoid was determined spectrophotometrically using the extinction co-efficiency of A^1%^_1cm_=2500, as proposed by Davis (14). Dry cell weight was determined at 105 ˚C to a constant weight. 

***Effect of time of incubation***

Samples were prepared as mentioned above by cultivation of the yeast strain on YPG and then transferring one full loop into YPG broth. Main cultures cultivation (MMS) was carried out in a 500 ml Erlenmeyer flask containing 100 ml of medium and was held at 30 ˚С in a shaker at 150 rpm for 24, 48, 72, 96, and 120 hr. All experiments were performed in triplicate. Extraction and quantification of carotenoids were carried out as outlined above.

***Statistical analysis***

All statistical analyses were performed using SPSS18 software (Statistical Package for Social Science). Data from the experiments were subjected to one-way analysis of variance (ANOVA) and student t-test. Values of *P*<0.001 were considered to be significant.  

**Table 1:** Amount of biomass and total carotenoid of two strains grown in 100 ml of MMS broth in a 500 ml Erlenmeyer flask incubated on a rotator shaker at 150 rpm and 30 ˚С for 72 hr

Strains	Dried cell mass (g l^-1^)	Carotenoid concentration(mg g^-1^)	Carotenoid production(mg l^-1^)
*Rhodotorula slooffiae*	3.325±0.035	5.4468±0.371*	0.9078±0.0619*
*Rhodotorula mucilaginosa*	3.85±0.212	0.8916±0.084	0.2972±0.028

The data are expressed as mean±SD. *P*<0.01  is considered significant

## Results

***Carotenoids analysis***

**Figure 1.**
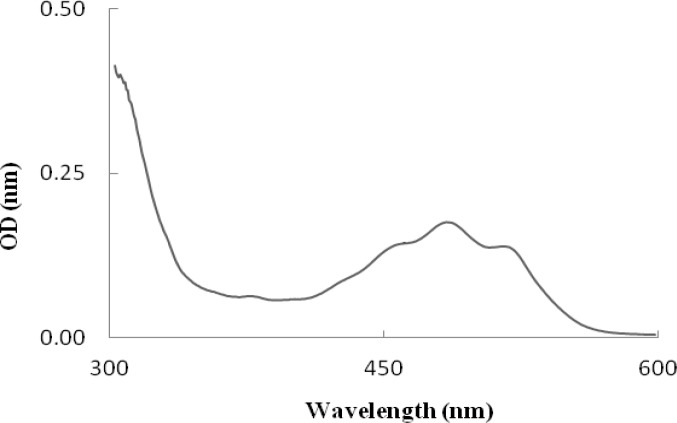
The spectrum of total carotenoid petroleum ether extract obtained from *Rhodotorula mucilaginosa*, with λ _max_= 457, 485, and 515 nm

**Figure 2:**
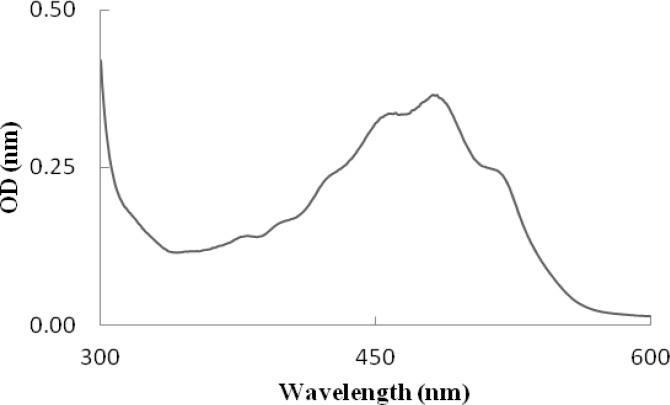
The spectrum of total carotenoid petroleum ether extract obtained from *Rhodotorula slooffiae*, with λ_max_= 457, 482, and 516 nm

The amount of biomass and total carotenoid are shown in Table 1. Both strains have the capacity to produce carotenoids. *R. slooffiae *significantly (*P*<0.001) presented higher level of carotenoid production and concentration compared to *R.* *mucilaginosa*, while the amount of cell growth in* R. mucilaginosa* was significantly (*P*<0.001) higher than *R. slooffiae*. Figures 1 and 2 show the spectra of the extract obtained from these strains. Each spectrum is typical of a mixture of carotenoids. The difference in shape indicates a difference in carotenoid composition. The maximum level in the spectra for *R. mucilaginosa *are recorded at 457, 485, and 515 nm but at 457, 482, and 516 nm for *R. slooffiae.* 

**Figure 3:**
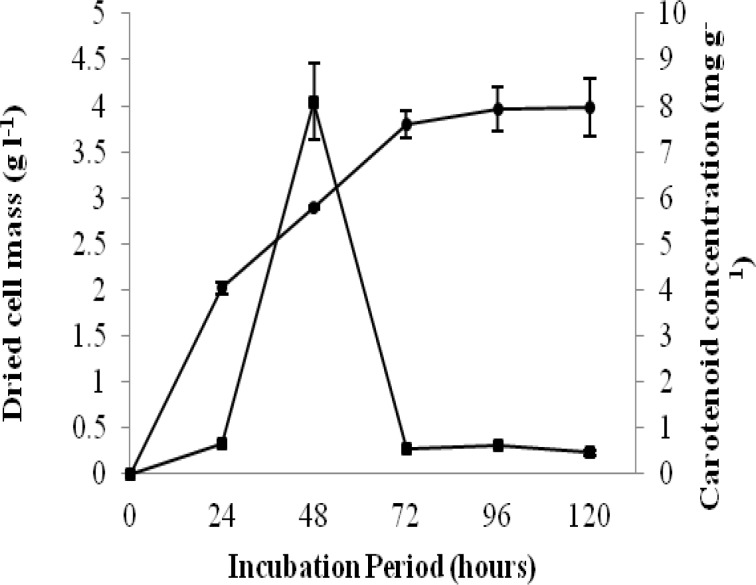
Growth and carotenoid production by *Rhodotorula mucilaginosa* at different incubation times, dried cell mass (g/l) [ ], Carotenoid concentration (mg/g)

[■]. Standard deviations are included. Values are means of 3 replicates ± SD.

**Figure 4:**
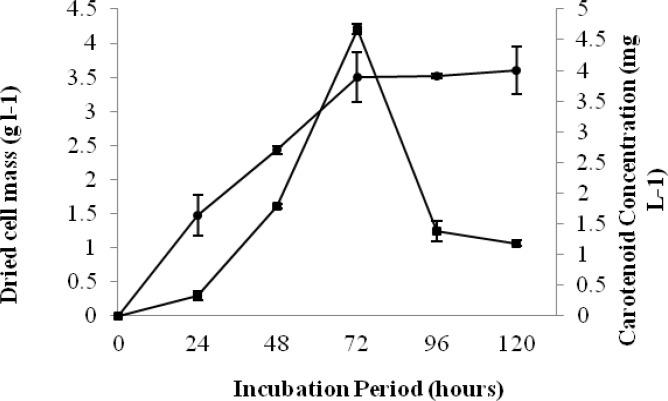
Growth and carotenoid formation by *R. slooffiae* at different incubation time, dried cell mass (g l^-1^) [ ], Carotenoid concentration (mg g^-1^)

***Effect of time of incubation***

Figures 3 and 4 show the time scale for carotenoid concentration and growth by the *R. mucilaginosa* and *R. slooffiae.* There was a significant (*P*<0.001) relationship between carotenoid and biomass concentration and incubation time in both strains. It was observed that the maximum cell mass accumulation and the maximum pigment accumulation do not occur at the same time. The results indicated that the dried cell mass in both strains significantly (*P*<0.001) increased along with rise of time. Following 120 hr, the maximum cell accumulation are 3.98±0.311 g l^-1^ and 3.60±0.353 g l^-1^ for *R. mucilaginosa *and *R. slooffiae,* respectively. 

As shown in Figure 3, in R*. mucilaginosa, *the carotenoid concentration reaches an optimum level after at 48 hr (8.094±0.845 mg/l) but reduces after 48 hr. *R. slooffiae* also shows an optimum for carotenoid concentration. Up to 72 hr, the carotenoid concentration of *R. slooffiae *increased (4.6728±0.084 mg/l) but after that, it decreased (Figure 4).

## Discussion

The results of comparing the amount of total carotenoid in two selected strains at 72 hr showed that *R. slooffiae *and* R. mucilaginosa *are differing in their carotenoid content and* R. slooffiae *possess a greater ability to produce carotenoids. However, the results of the effect of incubation time indicated that overall, *R. mucilaginosa *has more potential to produce carotenoid. The results of studies done byFrengova *et al* (15) and Martin *et al* (11) under various conditions on *R. slooffiae* were 2.67 mg/l (higher than our results) and 1.256 μg/g (lower than our results), respectively. In case of *R. mucilaginosa*, also the carotenoid concentration was higher than those reported for this strain by other researchers (16, 17). These studies also indicated that *R. Mucilaginosa *has the highest potential of carotenoid production among the strains employed in these studies, a fact that can also be confirmed by our data. It was reported that *R. mucilaginosa* will be one of the most promising microorganisms for the commercial production of carotenoids, by optimizing the culture condition (18). The difference between the values of carotenoid concentration in the two strains used our study with other researches may be explained by: [a] difference in environmental condition of experiments, [b] possibility of occurrence of genetic changes in strains and [c] efficient extraction method.

There are few works in literature that report the effects of incubation time on the carotenoid production. The results of our study reveals that *R. mucilaginosa *produces the highest value of carotenoid, when incubated for 48 hr. This result was different from that of Libkind *et al* (19). They demonstrated that the highest value of carotenoid is produced at 72 hr. Libkind *et al*, the same as our experience, observed negative correlation between pigment production and biomass accumulation. Buzzini *et al* (20), Fang and Chiou (21), Johnson and An (22) have shown that the maximum values of total carotenoids are not directly correlated with the maximum value of cell biomass. Voaides and Dima (23) and An *et al* (24) also have been observed increased growth of yeast cells as the time of incubation rises, which is in accordance with our data. Carotenoid pigments accumulation in most yeasts starts in the late logarithmic phase and continues in the stationary phase (25). Frengova *et al* (15) determined that the carotenoid contents in the cell mass reaches their maximum levels when the cell growth practically ends i.e., at an early stationary phase, a fact that can be confirmed also by the data obtained from *R. slooffiae* but not by the data from *R. mucilaginosa*. Similar data was reported about *R. slooffiae,* although with a different result regarding the amount of carotenoid, presumably due to the differences between experiments conditions (11). It was observed that the carotenoids are formed almost parallelly with the cell growth which did not support the correctness of our results, but in this study, they showed that different strains have different patterns of development and production of carotenoid, which was similar to our results for different strains of Rhodotorula (26). 

## Conclusion

Incubation periods were proved to have influence on the yeasts biomass accumulation and carotenoids production. The results suggested that the maximum rate of total carotenoids production is not directly associated with the maximum amount of cell biomass and the type of carotenoid and their relative amount may vary depending on genus of yeast. It seemed that carotenogenic yeasts do not exhibit a common pattern of cell growth and carotenoid production in response to different incubation periods. Even though the biosynthesis of carotenoids by *Rhodotorula* yeasts is well known, their industrial use is restricted mainly because of poor information on biosynthetic regulating mechanisms and lack of studies. It is obvious that both strains have ability to produce carotenoid. However, further studies are needed to clarify the use of this strain in food industry, pharmaceuticals and cosmetics. The search for optimal conditions of biomass and carotenoid production in these strains is now in progress in laboratory scale.
